# Resurgence of Diphtheria in North Kerala, India, 2016: Laboratory Supported Case-Based Surveillance Outcomes

**DOI:** 10.3389/fpubh.2017.00218

**Published:** 2017-08-30

**Authors:** Lucky Sangal, Sudhir Joshi, Shalini Anandan, Veeraraghavan Balaji, Jaichand Johnson, Asish Satapathy, Pradeep Haldar, Ramesh Rayru, Srinath Ramamurthy, Asha Raghavan, Pankaj Bhatnagar

**Affiliations:** ^1^WHO India, World Health Organization, New Delhi, India; ^2^Department of Clinical Microbiology, Christian Medical College, Vellore, India; ^3^State Public Health Laboratory, Thiruvananthapuram, India; ^4^WHO India, World Health Organization, Bangalore, India; ^5^Ministry of Health and Family Welfare, Government of India, New Delhi, India; ^6^Ministry of Health, Government of Kerala, Thiruvananthapuram, India; ^7^WHO India, World Health Organization, Kozhikode, India; ^8^WHO India, World Health Organization, Thiruvananthapuram, India

**Keywords:** vaccine preventable diseases, *Corynebacterium diphtheriae*, outbreak, molecular surveillance, multi-locus sequence typing

## Abstract

**Introduction:**

As part of national program, laboratory supported vaccine preventable diseases surveillance was initiated in Kerala in 2015. Mechanisms have been strengthened for case investigation, reporting, and data management. Specimens collected and sent to state and reference laboratories for confirmation and molecular surveillance. The major objective of this study is to understand the epidemiological information generated through surveillance system and its utilization for action.

**Methods:**

Surveillance data captured from reporting register, case investigation forms, and laboratory reports was analyzed. Cases were allotted unique ID and no personal identifying information was used for analysis. Throat swabs were collected from investigated cases as part of surveillance system. All *Corynebacterium diphtheriae* isolates were confirmed with standard biochemical tests, ELEK’s test, and real-time PCR. Isolates were characterized using whole genome-based multi locus sequence typing method. Case investigation forms and laboratory results were recorded electronically. Public health response by government was also reviewed.

**Results:**

A total of 533 cases were identified in 11 districts of Kerala in 2016, of which 92% occurred in 3 districts of north Kerala; Malappuram, Kozhikode, and Kannur. Almost 79% cases occurred in >10 years age group. In <18 years age group, 62% were male while in ≥18 years, 69% were females. In <10 years age group, 31% children had received three doses of diphtheria vaccine, whereas in ≥10 years, 3% cases had received all doses. Fifteen toxigenic *C. diphtheriae* isolates represented 6 novel sequence types (STs) (ST-405, ST-408, ST-466, ST-468, ST-469, and ST-470). Other STs observed are ST-50, ST-295, and ST-377.

**Conclusion:**

Diphtheria being an emerging pathogen, establishing quality surveillance for providing real-time information on disease occurrence and mortality is imperative. The epidemiological data thus generated was used for targeted interventions and to formulate vaccine policies. The data on molecular surveillance have given an insight on strain variation and transmission patterns.

## Introduction

The Universal Immunization Program (UIP) aims to reduce morbidity and mortality from vaccine preventable diseases (VPD) to levels that no longer constitute a public health problem. Laboratory supported VPD surveillance is an important tool for providing real-time information on the occurrence of VPDs and measures the progress made by the immunization program (1).

The state of Kerala launched laboratory supported case-based VPD surveillance, including diphtheria, with support from World Health Organization-National Polio Surveillance Project (WHO-NPSP) in April 2015. The suspected cases are reported by reporting network that includes both public and private sector health facilities. Mechanisms have been strengthened for case investigation, reporting, and data management. This system has been utilized for effective case management and public health interventions in response to identified cases and outbreaks of diphtheria.

Laboratory confirmation of clinically suspected diphtheria cases is crucial for accurately classifying cases and build confidence in the surveillance system. As part of the national program, capacity building and system strengthening has been done for two identified laboratories in Kerala state for performing laboratory diagnosis of diphtheria as per WHO norms. These are State Public Health Laboratory (SPHL), Thiruvananthapuram, and Microbiology department at Government Medical College, Kozhikode. A national reference laboratory responsible for standardization of laboratory procedures, technology transfer to other network laboratories, and quality assurance has been established at Christian Medical College (CMC), Vellore.

Numerous reports on the outbreak assessment and epidemiological surveillance of diphtheria from different parts of India have been previously reported (2–5). However, most of these reports are of data generated by various academic institutions as short-term research projects. The main objective of this study is to understand the epidemiological information generated through the surveillance system and how best it was utilized to take evidence-based public health measures. To the best of our knowledge, this is the first report on the diphtheria outbreak in Kerala along with molecular surveillance.

## Materials and Methods

We analyzed surveillance data available from the VPD surveillance system in the state of Kerala. The data sources were reporting register, case investigation forms, and laboratory reports.

As per standard VPD surveillance system protocol, cases were reported through a network of reporting sites. The case definition that was used for diphtheria surveillance was the standard WHO definition.

An illness of upper respiratory tract characterized by the following:

- Laryngitis or pharyngitis or tonsillitis,
- and adherent membranes of tonsils, pharynx, and/or nose.

All suspected cases that were reported to the system were investigated preferably within 48 h by trained medical officers. A unique identification number was allotted at district level to all such cases. The investigating officer or a trained health worker collected a throat swab specimen and send it to a WHO supported laboratory for culture and toxigenicity test.

The case investigation forms were recorded electronically by WHO-NPSP in software called Surveillance Information Management System that could be assessed real time at local, state, and national levels. The system recorded multiple variables like socio-demographic data, clinical history, treatment history, vaccination status, contact information, travel history, and type of sample taken.

The laboratory results were captured in software called Vaccine Preventable Disease Laboratory Information for Action and fed forwarded to national level. The case-based records and laboratory results were then electronically merged by an automated process based on unique identification number of the cases.

Identification of any diphtheria case in Kerala was followed by active case search in the community and contact tracing. Reporting of high number of diphtheria cases in the state had alerted the state government of Kerala for taking focused actions to curtail the further spread of diphtheria outbreaks. A steering committee, State Technical Advisory Group on Immunization (STAGI), was constituted to oversee the response and make appropriate recommendations. Other organizations of the private sector like Indian Medical Association, Indian Academy of Pediatrics, and religious heads were involved to spread awareness and early notification of diphtheria suspected cases. The cases were managed and public health interventions were taken as outlined in the VPD surveillance guidelines. We have reviewed the response of the government to diphtheria cases and recommendations of the steering committee.

This study was based on surveillance data available from national program and molecular typing of *Corynebacterium diphtheriae* isolates from routine specimen collection. It does not involve any participation of patients; hence, informed consent was not required. The study was in agreement with Government of India and State Government of Kerala. This study was approved by Institutional Research Board of CMC, Vellore at the meeting conducted on 28-10-2016 (IRB Min No: 9706).

### Laboratory Methods for Identification of Diphtheria

#### Culture

The throat swabs were processed and the isolate was identified by culture using 5% sheep blood agar and Serum Tellurite agar.*C. diphtheriae* colonies were confirmed with the Cystinase test growing black colonies with brown halo on Tinsdale agar (DIFCO, USA). The species was further confirmed with biochemical testing based on the utilization of glucose, dextrose, sucrose, maltose sugars followed by nitrate and urease tests (Fisher Scientific, MA, USA).

#### Toxigenicity Testing—Modified Elek’s Test

The toxigenicity testing was performed according to the previously reported method (6). Briefly, Elek’s agar medium with 20% newborn bovine serum was used to plate two test strains and three control strains on one plate. An antitoxin strip (500 U/ml, VINS Bioproducts Ltd., Hyderabad, India) was placed on the center of the plate and incubated at 37°C for 24 and 48 h. At 24 h using a suitable light source plates were observed for precipitin lines of identity between the test strains and the strong and weak positive control strains.

#### Real-time PCR Detection

DNA was extracted using QIAamp DNA blood mini kit (QIAGEN, Germany). The target genes include *rpoB* for *C. diphtheriae, C. ulcerans*, and *toxA* gene fragment *of C. diphtheriae*. Primers and conditions used in this study were previously reported for the Light Cycler (Roche, USA) PCR platform (Table 1) (7). This study tailored the similar protocol using the 7,500 Fast Real-time PCR (Applied Bio Systems, USA). Briefly, the PCR reaction set-up included 95°C for 10 min; followed by 45 cycles of 95°C for 15 s, and 60°C for 30 s. *C. diphtheriae* Ct cut off values for positivity were minimum 31.24 and maximum 34.06, for *C. ulcerans* minimum 28.96 and maximum 31.12, and for toxA minimum 31.05 and maximum 35.03.

**Table 1:** Real-time primers for the identification of *C. diphtheriae*.

Target	Primer/probe	Sequence 5′–3′	Amplicon length (bp)
*C. diphtheriae*	dip_rpobF	CGT TCG CAA AGA TTA CGG AAC CA	97
*rpo*B	dip_rpobR dip_probe	CAC TCA GGC GTA CCA ATC AAC HEXd-AGG TTC CGG GGC TTC TCG ATA TTC A-BHQ1	
*C. ulcerans* *rpo*B	ulc_rpobF ulc_rpobR ulc_probe	TTC GCA TGG CTC ATT GGC AC TCC AGG ATG TCT TCC AGT CC FAM-CCA GCA GGA GGA GCT GGG TGA A-BHQe1	98
Toxin	toxAF toxAR diptoxHP	CTT TTC TTC GTA CCA CGG GAC TAA CTA TAA AAC CCT TTC CAA TCA TCG TC ROX-AAG GTA TAC AAA AGC CAA AAT CTG GTA CAC-BHQ2	117

*The primer sequences used were previously reported (7)*.

#### Whole Genome Sequencing-Based Multi-Locus Sequence Typing (MLST)

Twenty-one lab confirmed isolates were subjected to whole genome sequencing by Ion torrent next generation sequencing technology as described in our previous study (8). Briefly, the whole genomic DNA was extracted as described before, and the purified DNA was used for the preparation of library fragments using the Ion Plus fragment library kit (Ion Torrent; Life Technologies, USA). Whole-genome sequencing was performed using the Ion Torrent PGM sequencer (Thermo Scientific Fisher Corp., USA) using 400-bp chemistry. The Raw sequence reads were assembled using SPAdes 5.0.0.0 tool embedded in Ion torrent server.

Multi-locus sequence typing profile of the isolates was predicted from the whole genome sequences by MLST 1.8 tool of the Centre for Genomic Epidemiology server (9). The allele loci of the unknown sequence types (STs) were submitted to PUBMLST database. Clonal analysis of the identified STs was performed by eBURST software (10, 11). The concatenated seven housekeeping gene sequences were aligned by Clustal-W and the phylogenetic tree was constructed using the Neighbor Joining algorithm by MEGA software. The evolutionary distances were computed by Jukes–Cantor method.

## Results

**Figure 1:**
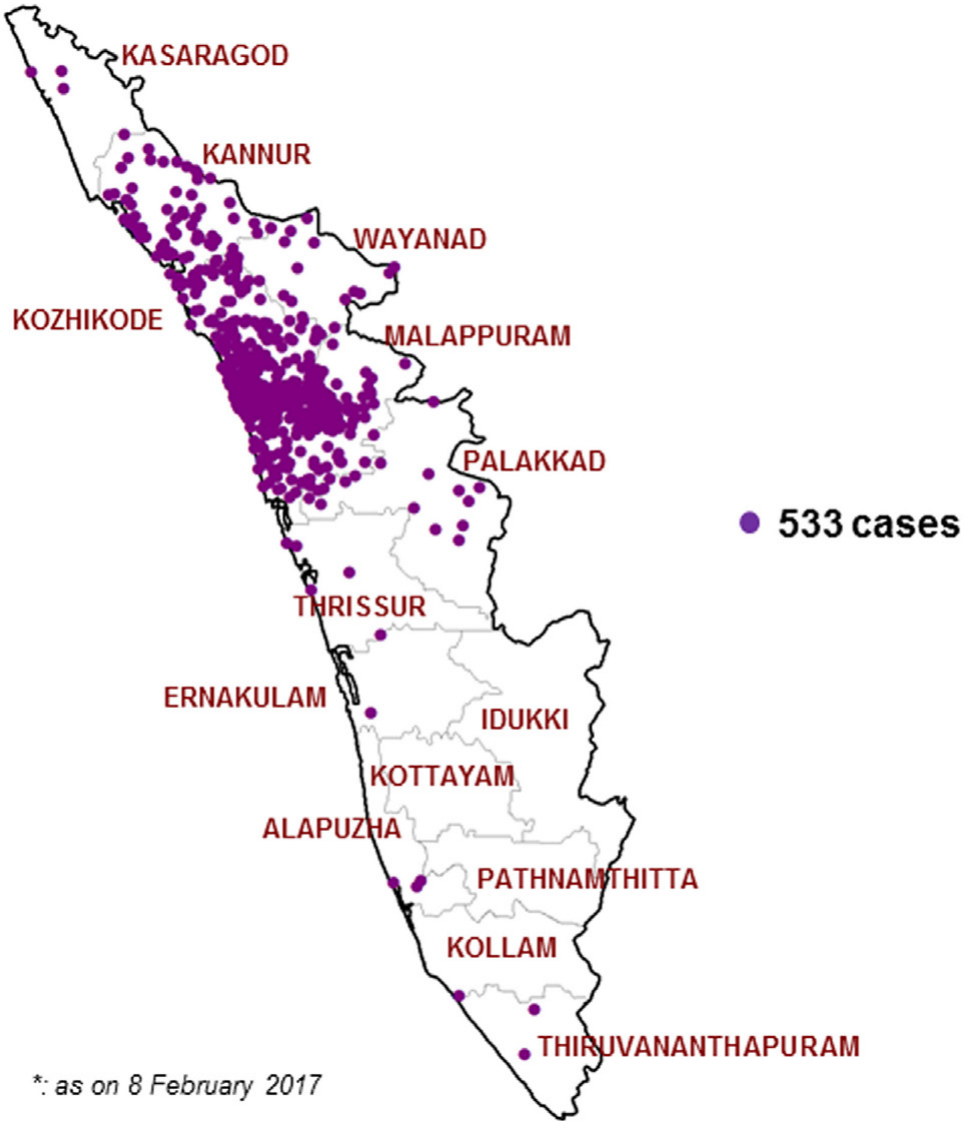
Map showing location of diphtheria cases in Kerala state, India 2016 (*n* = 533).

**Figure 2:**
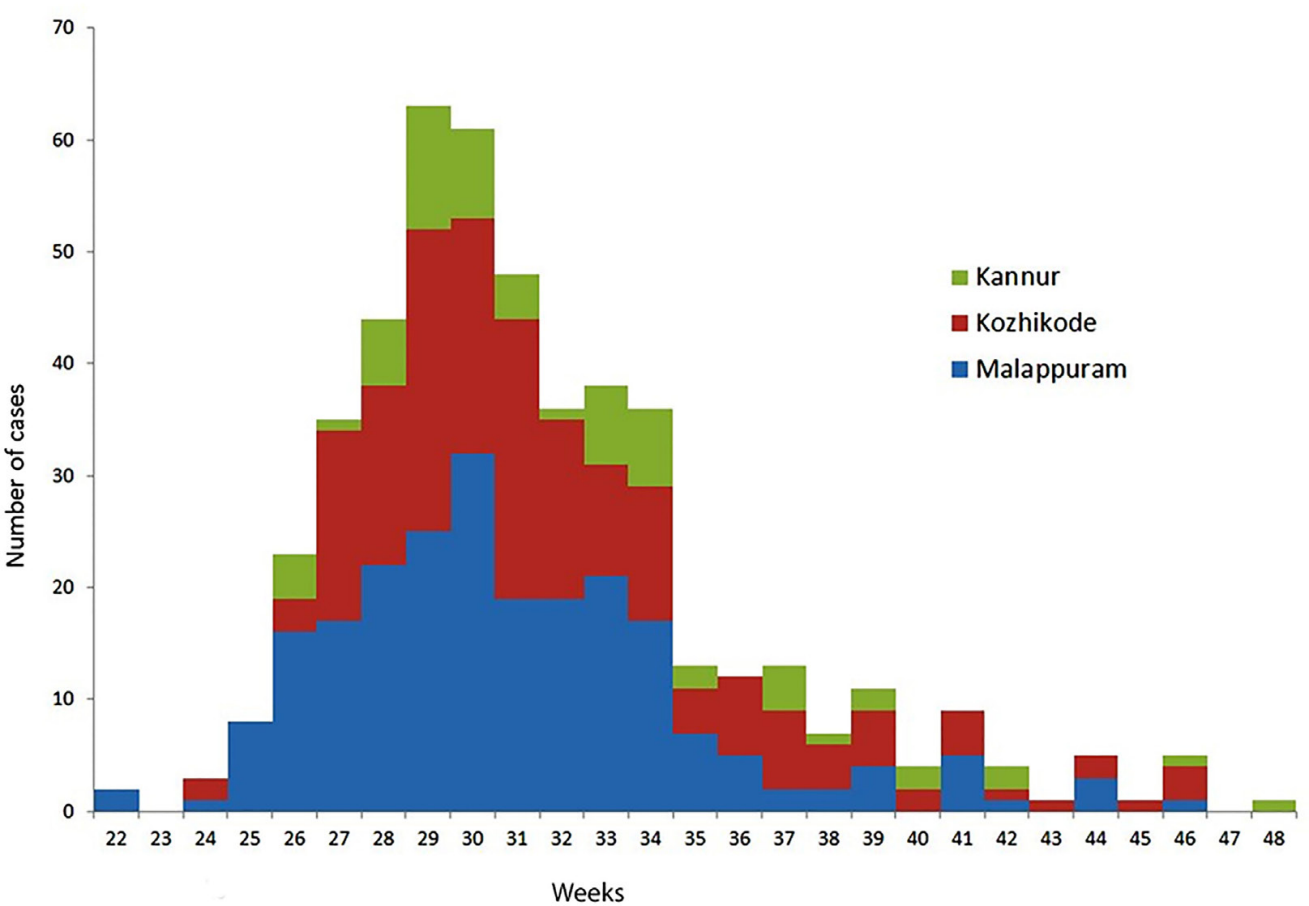
Epidemic curve of diphtheria cases in Malappuram, Kozhikode, and Kannur districts of Kerala, weeks 22–48; 2016 (*n* = 483).

After initiating the VPD surveillance in Kerala, 533 cases have been identified in 11 districts in 2016. A surge in reporting of suspected Diphtheria cases was noticed from May 2016 onward and a total number of 527 cases occurred in time period of 31 May 2016 to 30 November 2016. Figures 1 and 2 show the time and place distribution of these 527 cases. The first case, reported on 31 May, was from Tanur block of Malappuram district. Soon after the reporting of diphtheria cases increased in northern part of Kerala with maximum number of cases from Malappuram district (*n* = 229) followed by Kozhikode (*n* = 190), Kannur (*n* = 64), Wayanad (*n* = 16), Palakkad (*n* = 15), Thrissur (*n* = 4), Kasargod (*n* = 3). Six cases were reported from three districts of South Kerala, two each from Alappuzha, Ernakulam, and Thiruvananthapuram. Figure 2 shows the epi curve of Diphtheria cases for the three districts, Malappuram, Kozhikode, and Kannur, which have the highest number of cases. The typical bell-shaped curve is noticed for Malappuram and Kozhikode almost starting, reaching peak, and waning at the same time. The cases in Kannur started 3–4 weeks later and show multiple peaks in the same time duration.

Table 2 shows the age and sex distribution of 527 diphtheria cases. Most of the cases (79%) occurred in more than 10 years age group. Just 7% cases were children under 5 years while another 7% were in the age group of more than 45 years. The overall sex distribution of diphtheria cases is almost proportionate in males and females. However, in preschool and school going age group (0–18 years) males seem to be more affected (62%) and among all the male cases 71% are in this age group. In adult population (≥18 years) females were more affected (69%) and among all females 60% were in this age group.

**Table 2:** Age and sex distribution of diphtheria cases in Kerala, India (weeks 20–48).

Age group (in years)	Females	Males	Total
0–5	12	23	35
5–10	39	54	93
10–18	58	103	161
18–45	135	63	198
≥45	29	10	39
Total	273	253	526

The religion break up of diphtheria cases shows that 69% of total cases occurred in Muslims. The proportions of cases in Muslim community in Malappuram, Kozhikode, and Kannur are 83, 61, and 52%, respectively. Two cases of age 15 and 16 years died in Malappuram district of which one was laboratory confirmed Diphtheria (CFR = 0.4%).

We analyzed immunization with diphtheria containing vaccine in different age groups (Table 3). As per UIP schedule in India diphtheria antigen is given at 6, 10, and 14 weeks with two boosters at 16–24 months and 5–6 years of age. In children less than 5 years and in the age group of 5–10 years, 23 and 11% children, respectively, were found to be vaccinated against diphtheria as per age. In children less than 10 years, 31% children had received three doses of primary vaccination and 68% were either not vaccinated or their vaccination status was not known. In age group of above 10 years, 3% cases received fives doses of diphtheria containing vaccine and 7% were partially immunized.

**Table 3:** Vaccination status of diphtheria cases in Kerala, India (weeks 22–48) 2016 (*n* = 527).

Doses of diphtheria containing vaccine							
Age groups	0^a^	1	2	3	4	5	Total
0–5	24	0	0	4	7	0	35
5–10	63	1	1	4	15	10	94
10–18	135	7	3	4	4	8	161
18–45	192	0	1	1	2	2	198
>45	39						39

*^a^Cases with unknown immunization history were included as zero doses*.

Response to diphtheria cases: sudden increase of cases from week 22 onward, death of index case, and media reports put the state government at high alert. A total of approximately 575 480 doses of Td vaccine was procured by the state and stocked at Malappuram district for distribution to neighboring districts. Erythromycin prophylaxis was provided to close contacts as evident from action taken reports.

### Laboratory Identification of Diphtheria

The number of cases in relation to the positivity for the samples received from the different centers is represented in the Table 4. MLST analysis for 21 *C. diphtheriae* isolates from SPHL, Trivandrum revealed diversity among the STs. Nine STs ST-50, ST-295, ST-377, ST-405, ST-408, ST-466, ST-468, ST-469, and ST-470 were seen in the isolates, with majority of them belong to the new STs, ST-405 (*n* = 7) and ST-466 (*n* = 4), respectively. The STs ST-50, ST-295, ST-408, ST-468, ST-469, and ST-470 were seen in one isolate each and two isolates belonging to the unknown ST’s were submitted for inclusion in the *C. diphtheriae* PubMLST database.

**Table 4:** Laboratory confirmation of *C. diphtheriae* isolates by culture, ELEK’s test, and real-time PCR for *rpoB* and *tox-A* gene.

Centers	Specimen received	Isolate received	Culture positives	Elek’s test positives	Real-time PCR results	
2016					*C. diphtheriae* (*rpo*B)	*tox*A
Thiruvananthapuram, State Public Health Laboratory	Isolate	21	20	18	20	18
	Throat Swab	69	1	1	2	2
Calicut, ASTER MIMS	Isolate	1	1	1	1	1

**Figure 3:**
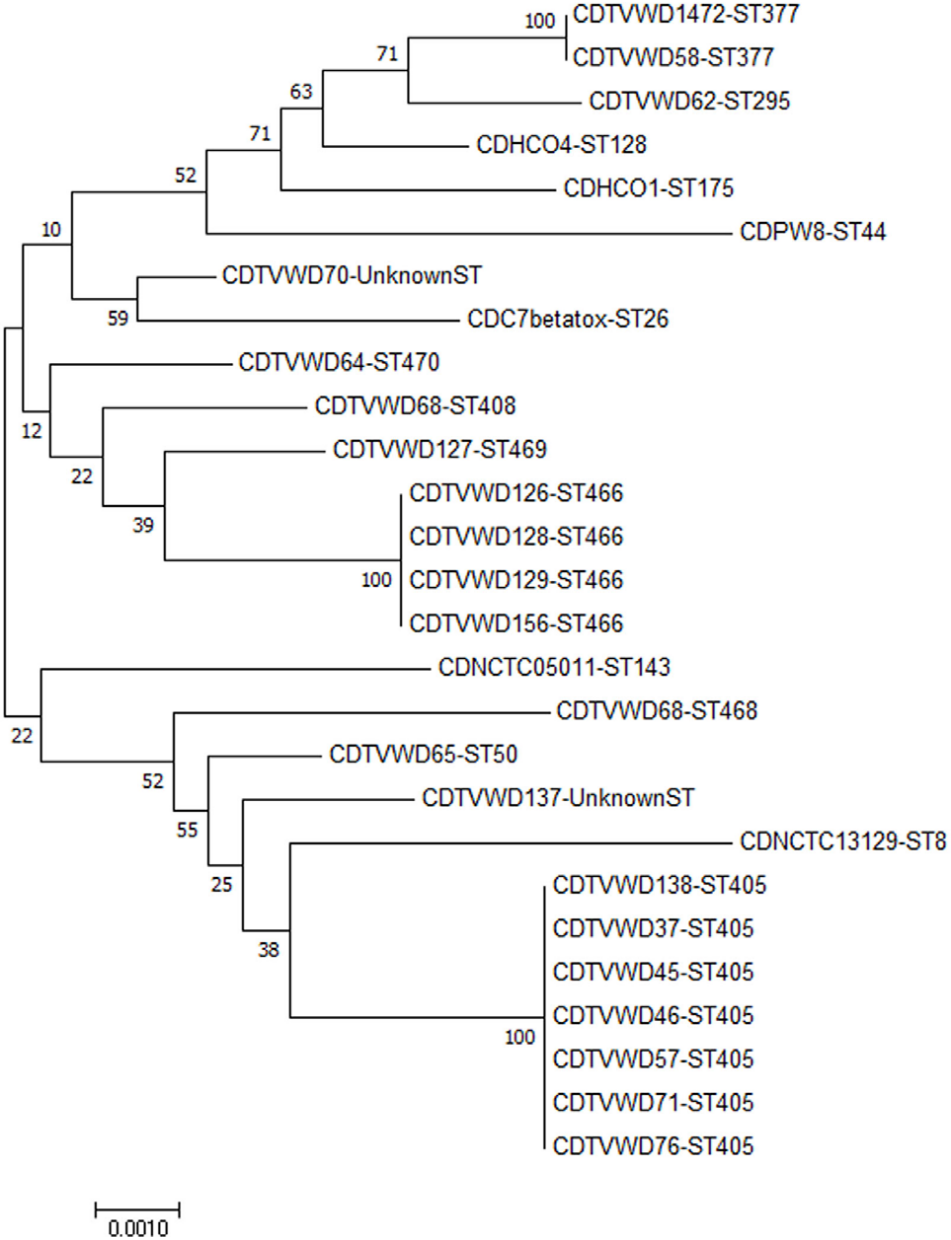
Phylogenetic tree including *C. diphtheriae* isolates from Kerala and reference strains from different parts of the world. The tree is represented by the isolate name followed by the sequence type.

**Figure 4:**
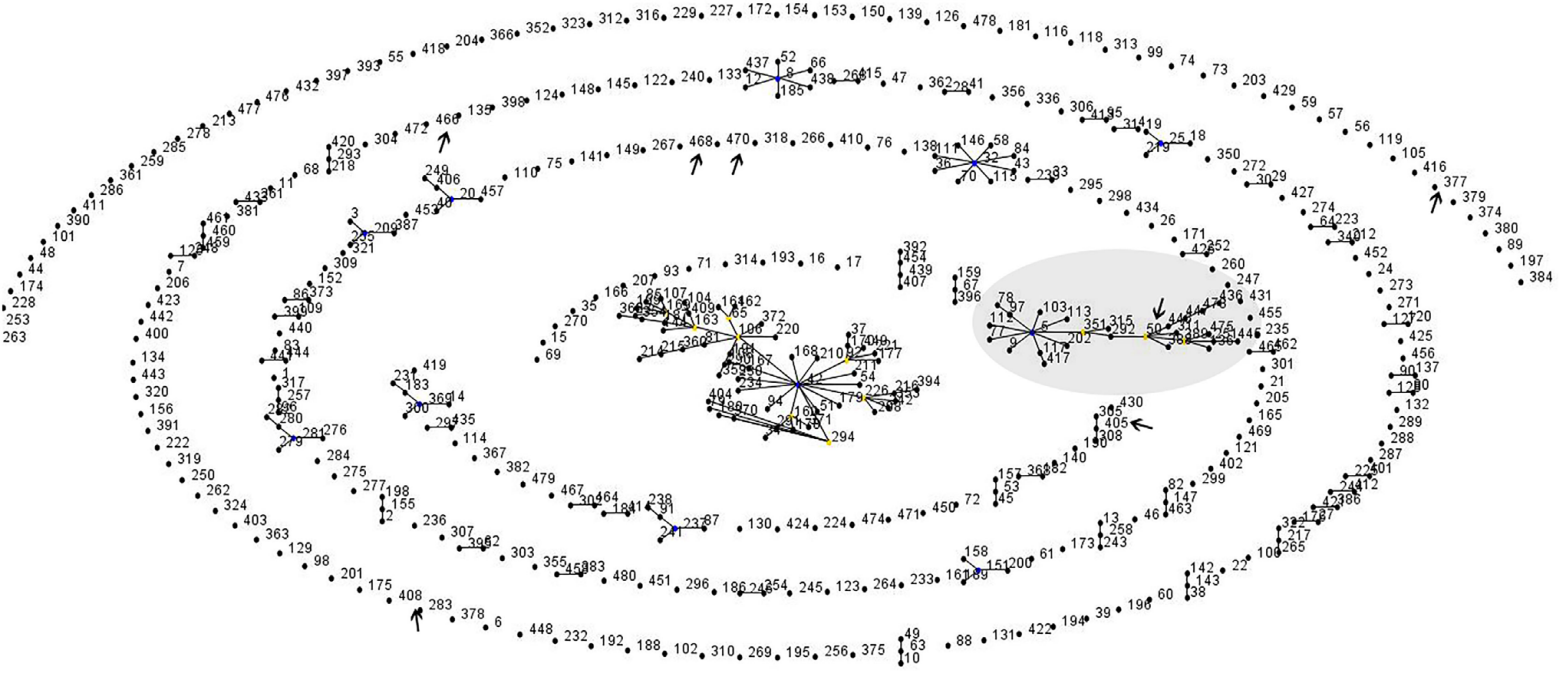
Global clonal relationship of the *C. diphtheriae* isolates predicted by eBURST analysis. Sequenced types observed in the Kerala outbreak were marked.

The phylogeny tree was constructed by neighbor joining algorithm for the MLST pattern derived from the allele loci of seven housekeeping genes obtained from the Kerala isolates and reference strains (NCTC 05011, HCO1, HC04, C7 beta^tox+^, NCTC 13129, INCA402) (Figure 3). The isolates WD1472, WD58, and WD 62 was closely related to the reference strains HC01 (ST-128), HC04 (ST-175), and PWD (ST-44). The PW8 strain was widely used for the production of diphtheria toxoid vaccine. The *C. diphtheriae* isolate WD 70 with unknown ST was closely related to the toxigenic strain C7 beta tox with ST ST-26. The six isolates with the new ST ST-405 was related to NCTC toxigenic strain 13129 (ST-8), which was isolated from the 2-year-old female with clinical diphtheria in United Kingdom. The NCTC 05011 strain originating from United Kingdom was found related to the isolates with STs ST-468, ST-50, and ST-405, respectively. The clonal relationship between the isolates from Kerala and other parts of the world is represented by eBURST diagram (Figure 4). eBURST analysis revealed the ST-50 was associated with the second major clonal complex (26 ST’s) with the founder ST-5. The newly reported ST in India, ST-405 forms complex with STs 305 and 308, respectively. The STs 295, 377, ST-408, ST-466, ST-469, and ST-470 do not belong to any clonal complexes circulating in world.

## Discussion

Of the total diphtheria cases reported in Kerala in 2016, 92% occurred in three districts of north Kerala; Malappuram, Kozhikode, and Kannur. The outbreak in this area could be due to the existence of pockets of low immunization coverage. Media reports have shown that vaccine acceptance is an issue noticed in certain sections of the community in north Kerala (12, 13).

The epi curve, from 22 to 48 weeks, shows that soon after the first case in Malappuram, the neighboring districts of Kozhikode and Kannur were largely affected. The epi curve of Kozhikode almost overlaps that of Malappuram suggesting that it was a part of the same event. The undulating curve of Kannur may be due to multiple exposures of susceptible population in this district over a time period of 25–43 weeks.

The surveillance data clearly indicates the shift in burden of diphtheria cases in adolescents and adults, as also noticed in other recently occurred Diphtheria outbreaks globally (14, 15). This could be due to traditional low immunization coverage in these pockets or waning of immunity after natural infection or any previous vaccination. Opportunities to provide immunization services to protect adolescents and adults should be encouraged, such as at school leaving age, pregnancy, etc. Tetanus toxoid (TT) vaccination is already a part of immunization schedule for adolescents (10 and 16 years of age) and pregnant women. Replacing TT vaccination with Td (TT and low dose diphtheria toxoid) vaccine has the potential to provide protection against diphtheria without any additional burden to the immunization program (16).

The sex distribution of cases shows that males are more affected during childhood or school going age group. However, after the age of 18 years, the females are significantly more affected. As women most commonly work in households and as caregivers in domestic settings, the chances of their exposure is high (14).

The religion break up of diphtheria cases shows that most of them are in Muslim community (69%). The Muslim population in Malappuram is high (70%) compared with rest of Kerala (27%), it is 39% in Kozhikode, and 29% in Kannur as per 2011 census. However, it is noticed that the proportion of diphtheria cases among Muslims are higher than the population proportion of Muslims in these three districts. It is 83, 61, and 52% in Malappuram, Kozhikode, and Kannur, respectively.

Kerala witnessed large number of diphtheria cases in 2016 despite traditionally achieving DTP3 coverage above 85% (87% in 2007–2008 and 93% in 2012–2013) (17, 18). The three districts of Kerala viz; Malappuram, Kozhikode, and Kannur that had witnessed large number of diphtheria cases in 2016 also had DTP3 coverage of 89, 97, and 95%, respectively, as per DLHS4. Certain sections of Muslim community in this area are reluctant to give vaccination to their children (12, 13) which might have led to pockets of susceptible cohorts. The government should target interventions in increasing vaccine acceptance among Muslims through involvement of religious leaders, community influencers, and educational institutions. These interventions should include booster doses because it has been seen that booster dose coverages fall leading to outbreaks (14). The polio eradication initiative in India has already proved that targeted interventions to increase vaccine acceptance in resistant communities reaps long term benefits to immunization program (19).

The early identification of diphtheria occurrence and death of two cases by the VPD surveillance system alerted the government to mount an early response. Constitution of STAGI and participation of both public/private sector effectively took appropriate and timely actions, like chemoprophylaxis and Td vaccination, to curtail the further spread of diphtheria in the community. Involvement of Department of Education was instrumental for Td vaccination in schools. The contact tracing at households and schools led to the early detection and management of additional cases resulting in low mortality and morbidity due to diphtheria. Diphtheria usually has a case fatality rate in the range of 5–10% (20) however, higher CFRs have been documented in many parts of the world. A diphtheria outbreak reported in Assam in 2015 documented 20% CFR (21).

The outbreak of diphtheria has been associated with concomitant periods of increase in the genomic diversity of the organism. The detailed investigation on the genomic information of the *C. diphtheria*e following the outbreak will help in analyzing the genetic diversity and transmission dynamics of the potential genes involved in virulence and development of resistance to antimicrobials. In recent years, advancement in next generation sequencing technologies has enabled fast and detailed investigation of the organism and help in identifying the clonality and transmission of the strain, involved in the infection. The application of appropriate typing methods is essential not only in outbreak investigations to monitor the evolution and spread of epidemic clones of *C. diphtheriae* but also in understanding and predicting epidemics.

The toxigenic strains sequenced in this study were characterized using traditional phenotypic and whole genome-based MLST typing methods. Seven of the toxigenic *C. diphtheriae* isolates (WD37, WD45, WD 46, WD57, WD71, WD76, and WD138) sequenced in this study represents the novel ST, ST-405, which was reported in our previous study isolates from Kadapa region of Andhra Pradesh (8). This implicates that same ST is circulating in two different states of South India (Andhra Pradesh and Kerala). The phylogram suggests the existence and persistence of a lineage that has been evolving and recurring in India, with special mention to the different districts in the state of Kerala. Two of the predicted STs (ST-405 and ST-50) associated with the outbreak in Kerala was related to clonal complexes circulating in the world, whereas the other STs are unique to the region.

## Conclusion

Laboratory supported VPD surveillance is an important tool for providing real-time information on the occurrence of VPDs and to take immediate actions to curtail the spread and reduce mortality. The surveillance data generated the evidence on changing epidemiology of diphtheria that helps the program not only to take targeted interventions but also to formulate vaccine policies. The current study in Kerala highlights the need for strengthening laboratory supported VPD surveillance across country. Further, the data on molecular surveillance of Diphtheria generated through laboratory support has given an insight on strain variation and transmission patterns. Further studies on the mechanisms of invasion, transmission dynamics of the disease, strain variation, and population structure of *C. diphtheriae* is necessary. Diphtheria being the emerging pathogen of increasing significance worldwide, it is imperative to take appropriate measures to control the disease.

## Informed Consent and Ethics

Since the study includes the surveillance and molecular typing of *C. diphtheriae* isolates, it does not involve participation of the patients. Hence, informed consent is not required. This study was approved by Institutional Research Board of Christian Medical College, Vellore at the meeting conducted on 28-10-2016 (IRB Min No: 9706).

## Author Note

In any use of this article, there should be no suggestion that WHO endorses any specific organization, products, or services. The use of the WHO logo is not permitted. This notice should be preserved along with the article’s original URL. The authors alone are responsible for the views expressed in this article and they do not necessarily represent the views, decisions, or policies of the institutions with which they are affiliated.

## Author Contributions

LS, SJ, SA, and VB conceived, designed the study. JJ, AS, SR, and AR conducted the surveillance. PH, RR, and PB organized and validated the study outcomes. LS, SJ, SA, and VB involved in computational analysis and drafting the manuscript. PH, RR, and PB reviewed and finalized the manuscript.

## Conflict of Interest Statement

The authors declare that the research was conducted in the absence of any commercial or financial relationships that could be construed as a potential conflict of interest.
